# Correlates of cocaine use during methadone treatment: implications for screening and clinical management (ANRS Methaville study)

**DOI:** 10.1186/s12954-016-0100-7

**Published:** 2016-04-05

**Authors:** Perrine Roux, Caroline Lions, Antoine Vilotitch, Laurent Michel, Marion Mora, Gwenaelle Maradan, Fabienne Marcellin, Bruno Spire, Alain Morel, Patrizia M. Carrieri, The ANRS Methaville study group

**Affiliations:** INSERM, UMR_S 912, Sciences Economiques & Sociales de la Santé et Traitement de l’Information Médicale (SESSTIM), 27 bd Jean Moulin, 13385 Marseille, France; Aix Marseille Université, UMR_S 912, IRD, Marseille, France; ORS PACA, Observatoire Régional de la Santé Provence Alpes Côte d’Azur, Marseille, France; INSERM, Research Unit 669, Paris, France; Univ Paris-Sud and Univ Paris Descartes, UMR-S0669, Paris, France; Centre Pierre Nicole, Paris, France; Oppelia, Paris, France

**Keywords:** Methadone, Opioid dependence, Cocaine, ADHD, Maintenance, Depression, Injection

## Abstract

**Background:**

Cocaine use is frequent in patients receiving methadone maintenance treatment (MMT) and can jeopardize their treatment response. Identifying clinical predictors of cocaine use during methadone treatment can potentially improve clinical management. We used longitudinal data from the ANRS Methaville trial both to describe self-reported occasional and regular cocaine use during MMT and to identify clinical predictors.

**Methods:**

We selected 183 patients who had data on cocaine (or crack) use at months 0 (M0), M6, and/or M12, accounting for 483 visits. The outcome was “cocaine use” in three categories: “no,” “occasional,” and “regular” use. To identify factors associated with the outcome over time, we performed a mixed multinomial logistic regression.

**Results:**

Time on methadone was significantly associated with a decrease in occasional but not in regular cocaine use from 14.7 % at M0 to 7.1 % at M12, and from 10.7 % at baseline to 6.5 % at M12, respectively. After multiple adjustments, opiate injection, individuals screening positive for attention deficit hyperactivity disorder (ADHD) symptoms, and those presenting depressive symptoms were more likely to regularly use cocaine.

**Conclusion**s**:**

Although time on MMT had a positive impact on occasional cocaine use, it had no impact on regular cocaine use. Moreover, regular cocaine users were more likely to report opiate injection and to present ADHD and depressive symptoms. Early screening of these disorders and prompt tailored pharmacological and behavioral interventions can potentially reduce cocaine use and improve response to MMT.

**Trial registration:**

The trial is registered with the French Agency of Pharmaceutical Products (AFSSAPS) under the number 2008-A0277-48, the European Union Drug Regulating Authorities Clinical Trials, number Eudract 2008-001338-28, the ClinicalTrials.gov Identifier: NCT00657397, and the International Standard Randomised Controlled Trial Number Register ISRCTN31125511.

## Background

Opioid maintenance treatment (OMT), especially methadone maintenance treatment (MMT), is widely recognized as a gold standard for managing opioid dependence [1]. However, many socio-economic, behavioral, and clinical conditions and determinants may impair response to MMT and consequently negatively impact the primary goal of this treatment which is abstinence from street-opioid use. Studies have pointed out that many opioid-dependent individuals are also either cocaine-dependent or are cocaine users [2–4] and that the effectiveness of methadone treatment is lower in this population [5]. Moreover, no pharmacological treatment currently exists for harmful cocaine use or dependence. Treatment is based on psychological intervention using cognitive behavioral therapy (CBT) or contingency management [6, 7].

Some studies have shown that retention in OMT has a positive impact on cocaine use, especially in less severe cocaine users [4, 8, 9]. However, other studies highlight that people who continue to use cocaine while on OMT are more likely to have a poorer response to treatment, in terms of retention and abstinence from street-opioid use [10, 11], and may also have a lower level of pharmacokinetic exposure to methadone, the consequence being a decreased effect of methadone [12]. Moreover, studies on methadone-related mortality [13, 14] have shown that cocaine use while on OMT is associated with a higher risk of overdose. A recent study conducted in San Francisco showed that more than one third of all fatal opioid overdoses involved also cocaine [15]. As it is known that cocaine use during methadone treatment may be associated with premature discharge [16], it seems important to identify predictors of cocaine use, in order to improve clinical management of these patients. Some hypotheses have been put forward regarding the possible association between psychiatric comorbidities—such as depression [17], attention deficit/hyperactivity disorder (ADHD) [18], alcohol dependence [19], and severe opioid dependence [20]—and more severe patterns of cocaine use [21, 22]. However, it would seem more relevant to investigate correlates of cocaine use more thoroughly and to provide clinicians with indications as to how they can better manage MMT patients whose cocaine use is deemed “at-risk.” We used longitudinal data from the ANRS Methaville trial, which enrolled opioid-dependent individuals starting methadone maintenance, to describe the pattern of cocaine use during MMT and to identify clinical correlates.

## Methods

### Study design

From January 2009 to January 2010, the ANRS Methaville study—a multi-site, open-label, randomized, controlled, non-inferiority trial—recruited 195 men and women in 10 sites in France. The study aimed to compare methadone initiation in France in specialized centers (standard care) with initiation in primary care. This study was approved by the ethics committee for the protection of patients in Paris, France. All individuals provided written, informed consent before participating in the study. The full protocol is described elsewhere [23]. Each participant was followed up for 12 months. Medical visits, completion of self-administered questionnaires, and phone interviews occurred at enrolment (M0), 3, 6, and 12 months (M3, M6, and M12, respectively).

### Variables and instruments

Computerized assisted phone interviews (CATI) were used to collect the following information at enrolment and during follow-up visits: (1) socio-demographic characteristics: age, gender, employment status, housing status (renter or owner); (2) history of drug use: age at first drug use, history of overdoses, history of drug injection; (3) current drug, alcohol, and tobacco use; (4) perception of the adequacy of the prescribed methadone dose, categorized into 3 possible answers: too high, adequate, too low. This was done to assess each interviewee’s satisfaction with methadone treatment.

Patients reporting that they had used cocaine or crack use once during the previous month were considered “occasional cocaine users,” while those reporting cocaine (or crack) use at least twice in the previous month were considered “regular cocaine users.”

Alcohol consumption assessment was based on the alcohol use disorders test (AUDIT) with a threshold of 13 indicating alcohol dependence [24], while depressive symptoms were assessed using the Center for Epidemiologic Studies-Depression Scale (CES-D) with a threshold of 17 indicating depression for males and 23 for females [25]. The Fagerstrom Scale, comprising 6 items, evaluated tobacco dependence, a threshold of 3 (on a 0 to 10 scale) defining dependence [26].

Drug use was assessed using the Opiate Treatment Index (OTI) questionnaire, which documented the previous 3 days when drugs were used (last 3 days when drugs were taken and amounts consumed) during the previous month [27].

The self-administered questionnaire at M0, M6, and M12 included two screening tools. The first was the Adult ADHD Self-Report Scale-Version 1.1 (ASRS-V1.1) which evaluates attention deficit hyperactivity disorder (ADHD) in patients. The scoring algorithm used was the sum score obtained adding up the scores (0–4) of the first 6 items [28]. Then, we defined the diagnosis of ADHD using 14 as a cut-off score [29]. The second tool was the Beck Hopelessness Scale (BHS), a 20-item self-reported inventory, where a score of 9 or more indicates suicide risk [30].

During medical visits, physicians collected data on withdrawal symptoms using the Objective Opioid Withdrawal Scale (OOWS) which comprises a list of 13 withdrawal symptoms [31].

During the phone interviews, patients were asked about their prescribed methadone dose. At each follow-up visit, physicians noted the methadone dose prescribed to the patient in the medical questionnaire.

### Statistical analyses

From the 195 patients included in the trial, we used data of 183 patients (177 at baseline) who answered the OTI section about cocaine use at least once during follow-up, accounting for 486 visits. For these analyses, we used only the M0, M6 and M12 visits where data for most of the variables were available. First, we compared the individual characteristics of the study sample between those who consumed cocaine or crack at enrolment (i.e. before methadone initiation) and those who did not. These comparisons were performed using a chi-squared test (for categorical variables) and a Wilcoxon test (for continuous variables). To identify the factors associated with cocaine use during treatment, we used a multinomial mixed model with a 3-category outcome: no cocaine use (reference), occasional cocaine use, and regular cocaine use.

We tested the following factors as possible explanatory variables for the consumption of cocaine: (1) socio-demographic characteristics (sex, age, employment, housing (home owner or renter)); (2) drug use-related factors (history of drug injection, previous drug overdose, opioid injection; (3) clinical factors: methadone-associated characteristics (methadone induction arm, methadone dose: ≥60 mg vs. <60 mg, perceiving methadone dose to be too low vs. adequate or too high); clinical factors (depressive symptoms, risk of suicide, ADHD symptoms, at least 1 withdrawal symptom); tobacco dependence and alcohol dependence. We first performed a univariate mixed multinomial logistic regression adjusted on methadone treatment duration. A liberal *p* value of <0.20 in the univariate analyses was chosen to select eligible variables for the multivariate model. A stepwise procedure was used to identify the best model by removing variables one at a time based on a *p* value of >0.05. All analyses were performed using the SPSS v15.0 (SPSS, Inc, Chicago, IL) and Intercooled Stata 12 (StataCorp LP, College Station, TX) software packages using the GLLAMM procedure for multinomial analysis.

## Results

### Sample description

Among the 177 patients with available data at enrollment, 29 (16.4 %) were female and median [IQR] age was 32 [27–38] years (Table 1). At the baseline visit, i.e. before starting methadone treatment, half of the patients (50.9 %) were employed and two thirds (62.1 %) rented or owned their home. With respect to psychiatric comorbidities, at the baseline visit, 39.4 % of the patients had depressive symptoms, 30.9 % had risk of suicide, and 32.2 % had ADHD symptoms. Sixty-two percent of the patients had at least one withdrawal symptom at the baseline visit. At the baseline visit, 13.9 % reported alcohol dependence, half reported a history of drug injection, and 12.4 % reported overdosing at some point in their life. Only 1.3 % of patients received more than 60 mg of methadone a day. Finally, 25.4 % reported cocaine use at baseline. At baseline, patients who reported using cocaine were significantly more likely to be unemployed, to have depressive symptoms, a risk of suicide, a history of drug injection and ADHD symptoms, than those who did not use cocaine (Table 1).

**Table 1:** Description of the sample: socio-demographic and behavioral characteristics at baseline

	No cocaine use (*n* = 132)	Occasional cocaine use (*n* = 26)	Regular cocaine use (*n* = 19)	Total (*n* = 177)	*p* value^a^
Gender, *n* (%)					
Male	110 (83.33)	23 (88.46)	15 (78.95)	148 (83.62)	0.70
Female	22 (16.67)	3 (17.54)	4 (21.05)	29 (16.38)	
Methadone induction arm					
Specialized centers	31 (23.5)	7 (26.9)	4 (21.1)	42 (23.7)	0.89
Primary care	101 (76.5)	19 (73.1)	15 (78.9)	135 (76.3)	
Age^b^, median (IQR)	32 (27–38)	30 (27–37)	32 (27–40)	32 (27–38)	0.77
Employed, *n* (%)					
No	55 (44.35)	15 (57.69)	13 (68.42)	83 (49.11)	0.09
Yes	69 (55.65)	11 (42.31)	6 (31.58)	86 (50.89)	
Home owner or renter, *n* (%)					
No	43 (34.68)	10 (38.46)	11 (57.89)	64 (37.87)	0.15
Yes	81 (65.32)	16 (61.54)	8 (42.11)	105 (62.13)	
Depressive symptoms, *n* (%)^c^					
No	80 (66.67)	12 (46.15)	8 (42.11)	100 (60.61)	0.03
Yes	40 (33.33)	14 (53.85)	11 (57.89)	65 (39.39)	
Risk of suicide, *n* (%)^d^					
No	79 (73.15)	16 (69.57)	8 (44.44)	103 (69.13)	0.05
Yes	29 (26.85)	7 (30.43)	10 (55.56)	46 (30.87)	
ADHD symptoms, *n* (%)					
No	76 (70.37)	17 (73.91)	8 (44.44)	101 (67.79)	0.07
Yes	32 (29.63)	6 (26.09)	10 (55.56)	48 (32.21)	
Previous drug overdose, *n* (%)					
No	117 (88.64)	22 (84.62)	16 (84.21)	155 (87.57)	0.64
Yes	15 (11.36)	4 (15.38)	3 (15.79)	22 (12.43)	
History of drug injection, *n* (%)					
No	66 (53.66)	12 (46.15)	5 (26.32)	83 (49.40)	0.08
Yes	57 (46.34)	14 (53.85)	14 (73.68)	85 (50.60)	
Age at first drug use, median (IQR)^b^	18 (17–22)	18 (16–21)	18 (17–19)	18 (17–21)	0.23
At least one withdrawal symptom, *n* (%)					
No	49 (42.24)	6 (25.00)	5 (26.32)	60 (37.74)	0.16
Yes	67 (57.76)	18 (75.00)	14 (73.68)	99 (62.26)	
Methadone dose >60 mg, *n* (%)					
No	110 (99.10)	20 (100.00)	17 (94.44)	147 (98.66)	0.25
Yes	1 (0.90)	0	1 (5.6)	2 (1.34)	
Alcohol dependence, *n* (%)^a,e^					
No	108 (88.52)	21 (84.00)	14 (73.68)	143 (86.14)	0.21
Yes	14 (11.48)	4 (16.00)	5 (26.32)	23 (13.86)	

^a^Chi-squared or exact Fisher test/Kruskal–Wallis

^b^In years

^c^CES-D score >17 for males and >23 for females

^d^Beck ≥9

^e^AUDIT score ≥13

### Cocaine use during the first 12 months of methadone maintenance treatment

**Fig. 1:**
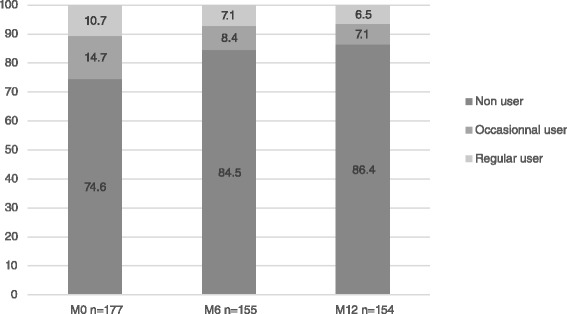
Cocaine use in methadone patients: M0, M6, and M12 visits

Figure 1 shows the percentage of patients reporting cocaine use at each visit: baseline (M0), M6, and M12. At the baseline visit, of those patients who had data on cocaine use (*n* = 177), 25.4 % reported using cocaine during the previous month, 14.7 % occasionally and 10.7 % regularly. The M6 visit showed a decrease in cocaine use with only 15.6 % patients reporting they used it (8.4 % occasionally and 7.1 % regularly) and this decrease remained stable at M12 with 13.6 % (7.1 % occasionally and 6.5 % regularly). Compared with enrolment therefore, the proportion of patients reporting cocaine use decreased significantly at the M6 and M12 visits (Table 2). Among the 45 cocaine users at baseline, 8 were lost to follow-up, 10 continued to use cocaine, and 27 stopped using it.

**Table 2:** Factors associated with cocaine use: logistic mixed model adjusted for follow-up visit

			Occasional cocaine use vs no use		Regular cocaine use vs no use	
	Number of visits (%) or median (IQR)	Number of patients	OR [95 % CI]	p	OR [95 % CI]	*p*
Follow-up						
M0	177 (36.4)	177 (96.7)	1		1	
M6	155 (31.9)	155 (84.7)	0.35 (0.15–0.82)	0.02	0.41 (0.16–1.01)	0.05
M12	154 (31.7)	154 (84.2)	0.28 (0.12–0.69)	0.01	0.35 (0.14–0.90)	0.03
Methadone induction arm						
Specialized centers	130 (20.5)	43 (23.4)	1		1	
Primary care	503 (79.5)	141 (76.6)	0.87 (0.28–2.69)	0.80	1.28 (0.37–4.40)	0.70
Gender						
Male	411 (84.6)	154 (84.2)	1		1	
Female	75 (15.4)	29 (15.8)	0.49 (0.12–2.10)	0.33	1.10 (0.29–4.23)	0.89
Age^a^	32 (27–39)		0.89 (0.46–1.73)	0.74	1.03 (0.52–2.02)	0.94
Employed						
No	131 (41.7)	99 (56.9)	1			
Yes	183 (58.3)	122 (70.1)	1.02 (0.43–2.39)	0.97	0.52 (0.21–1.30)	0.16
Home owner or renter						
No	107 (34.1)	76 (43.7)	1		1	
Yes	207 (65.9)	128 (73.6)	0.77 (0.31–1.92)	0.57	0.33 (0.13–0.86)	0.02
Previous drug overdose						
No	429 (88.3)	161 (88.0)	1		1	
Yes	57 (11.7)	22 (12.0)	2.07 (0.51–8.33)	0.31	2.30 (0.55–9.57)	0.25
History of drug injection						
No	244 (51.6)	88 (50.6)	1		1	
Yes	229 (48.4)	86 (49.4)	1.99 (0.75–5.30)	0.17	3.98 (1.39–11.37)	0.01
Age at first drug use^a^	18 (17–21)		0.92 (0.83–1.02)	0.12	0.86 (0.75–0.98)	0.03
Methadone dose >60 mg						
No	305 (66.7)	164 (92.7)	1		1	
Yes	152 (33.3)	84 (47.5)	1.37 (0.51–3.68)	0.54	0.82 (0.29–2.32)	0.71
Perceiving methadone dose to be too low						
No	265 (94.3)	151 (96.8)	1		1	
Yes	16 (5.7)	13 (8.3)	1.23 (0.08–18.09)	0.88	6.06 (0.68–53.78)	0.11
Depressive symptoms^b^						
No	310 (68.3)	142 (81.6)	1		1	
Yes	144 (31.7)	86 (49.4)	2.12 (0.88–5.11)	0.09	4.32 (1.71–10.92)	0.002
Risk of suicide^c^						
No	279 (72.7)	133 (81.1)	1		1	
Yes	105 (27.3)	69 (42.1)	1.62 (0.68–3.86)	0.28	3.93 (1.59–9.76)	0.003
ADHD symptoms						
No	304 (79.0)	148 (89.7)	1		1	
Yes	81 (21.0)	59 (35.8)	1.48 (0.55–4.01)	0.44	6.17 (2.34–16.22)	<0.001
At least 1 withdrawal symptom						
No	296 (72.0)	148 (87.1)	1		1	
Yes	115 (28.0)	103 (60.6)	1.42 (0.52–3.89)	0.50	1.51 (0.52–4.38)	0.45
Alcohol dependence^d^						
No	274 (88.1)	157 (91.3)	1		1	
Yes	37 (11.9)	27 (15.7)	3.08 (0.88–10.80)	0.08	4.10 (1.15–14.61)	0.03
Opiate injection^e^						
No	414 (91.4)	164 (95.3)	1		1	
Yes	39 (8.6)	24 (13.8)	6.06 (1.29–28.44)	0.02	12.42 (2.72–56.61)	0.001

Univariate analyses: *N* = 486 visits; 183 persons

^a^In years

^b^CES-D score >17 for males and >23 for females

^c^Beck ≥9

^d^AUDIT score ≥13

^e^During the previous 4 weeks

### Factors associated with occasional cocaine use

Univariate analysis (Table 2) highlighted the eligibility of several variables for the multivariate model (*p* < 0.20). First, no socio-demographic variable was associated with occasional cocaine use. A history of drug injection, current opioid injection, depressive symptoms, and alcohol dependence were considered eligible variables to explain occasional cocaine use. A significant decrease in occasional cocaine use was observed as the duration of methadone treatment increased.

After multivariate analysis (Table 3), two variables remained positively associated with the outcome: a longer duration on methadone treatment (at the M12 visit only) was associated with less occasional cocaine use and current opioid injection was associated with higher risk of being occasional cocaine user. Those who had depressive symptoms were slightly more likely to be occasional cocaine users.

**Table 3:** Factors independently associated with occasional and regular cocaine use: logistic mixed model

	Occasional cocaine use vs no use		Regular cocaine use vs no use	
	ORa [95 % CI]	*p*	ORa [95 % CI]	*p*
Follow-up				
M0	1		1	
M6	0.48 [0.18–1.27]	0.14	0.77 [0.26–2.26]	0.63
M12	0.33 [0.12–0.95]	0.04	0.38 [0.11–1.32]	0.13
Depressive symptoms^b^				
No	1			
Yes	2.41 [0.92–6.31]	0.07	3.49 [1.21–10.04]	0.02
ADHD symptoms				
No	1			
Yes	1.40 [0.47–4.18]	0.55	5.23 [1.74–15.71]	0.003
Opiate injection^a^				
No				
Yes	5.73 [1.26–25.99]	0.02	8.98 [1.87–43.22]	0.01

Multivariate analyses: *N* = 353 visits; 156 persons

^a^During the previous 4 weeks

^b^CES-D score >17 for males and >23 for females

### Factors associated with regular cocaine use

For regular cocaine use, univariate analysis highlighted the same variables as those for occasional cocaine use. However, additional variables were associated with regular cocaine use. With respect to socio-demographic variables, regular cocaine users were less likely to be owners or renters of their house and to be employed. In addition, perceiving methadone dose as too low, suicidal risk and ADHD symptoms were also eligible to enter the multivariate model. After the latter was analyzed, three variables remained associated with regular cocaine use: depressive symptoms, ADHD symptoms and current opioid injection. It is worth noting that time on methadone was not associated with a reduction in regular cocaine use.

The methadone induction arm (specialized center versus primary care) was not associated with either occasional or regular cocaine use.

## Discussion

Our results showed that cocaine use was highly prevalent among opioid-dependent individuals initiating methadone maintenance treatment, with almost one third of the sample reporting it at baseline. Furthermore, depressive symptoms, ADHD disorders, and current opioid injection were the main predictors of regular cocaine use. These results may have important repercussions on clinical management of these patients.

Our results about cocaine use prevalence are in line with other studies where 30 to 50 % of opioid-dependent individuals seeking care for opioid dependence used cocaine concomitantly [32–34]. More specifically, in our study, at baseline, 14.7 and 10.7 % of the study sample were occasional and regular cocaine users, respectively. This is in line with other findings [35].

Cocaine use decreased during the 12-month methadone maintenance treatment (MMT) follow-up, with 27 (15 %) patients stopping use altogether. This result corroborates findings in other studies [9, 36] and suggests that despite treatment, certain cocaine users do not reduce their cocaine consumption. In turn, this may act as a barrier to MMT optimization. Accordingly, these vulnerable patients deserve special investigation, particularly those who continue to use cocaine regularly.

To better understand patterns of cocaine use in MMT patients, we separated occasional users from regular users at all visits and studied the factors associated with occasional and regular cocaine use over 12 months of follow-up. Univariate analyses revealed that patients with the most severe characteristics, that is to say, users with a longer history of drug use, those with a history of injecting practices and polydrug users, were more likely to use cocaine during MMT.

One of the main results from the present study is that ADHD was associated with regular cocaine use. It is known that ADHD is a common psychiatric comorbidity among cocaine-dependent patients [37], as cocaine may be used as a means of self-medication for the disease [38]. Furthermore, it is well known that substance use and ADHD are closely correlated [39]. Consequently, screening for ADHD at methadone initiation and appropriate clinical management during methadone treatment, together with the monitoring of cocaine use, can potentially help patients to improve their response and reduce overdose risks [13, 14]. Few clinical responses currently exist for cocaine dependence [40] and cocaine use during MMT [41–43]. Standard treatment includes contingency management and cognitive behavioral therapy [6, 44, 45]. However, findings regarding the efficacy of psychotherapy for cocaine abuse in MMT patients are mixed. For example, in one article, counseling therapy for cocaine users in a population of methadone-maintained patients was not effective [46], while another article showed that CBT may indeed have a positive impact [6]. More generally, long-term studies highlight the weakness in providing only CBT [47].

We also found that depressive symptoms were associated with cocaine use. The relationship between cocaine use and depression is complex, and the causality direction is unclear. It is known that cocaine-dependent individuals are more likely to have depressive symptoms, depression perhaps being a preliminary condition for cocaine dependence [48]. However, one recent study has also highlighted that depression may also be a consequence of cocaine use [49]. In any case, depression should be diagnosed and treated in a timely fashion in order to reduce its negative impact on the effectiveness of OMT. Although it has been shown that certain anti-depressants are not effective in reducing cocaine use in cocaine and opioid co-dependent patients [50, 51], some studies have demonstrated that access to adequate care for depression in cocaine-dependent patients may lead to decreased cocaine use [52, 53].

In addition, persistent opiate injection during methadone maintenance (i.e., non-response to opioid dependence treatment) was associated with persistent (both occasional and regular) cocaine use. This suggests that methadone treatment (at the prescribed dosages) may be an inadequate opioid dependence treatment; and that for such patients, methadone treatment has a negligible impact on cocaine use. In addition, two associations with regular cocaine use found in the univariate analyses may help us better understand the relationship between heroin injection and regular cocaine use: reporting withdrawal symptoms and a tendency to perceive methadone dose as too low were both associated with regular cocaine use (but not occasional cocaine use). This may also suggest that cocaine has a pharmacokinetic impact on methadone which decreases the effect of the latter [12]. To conclude, persistent heroin injection during MMT may be explained by underdosing of methadone [54] and/or inappropriate treatment for opioid dependence [55].

Finally, it is interesting to note that a longer time on methadone (measured in terms of each follow-up visit) was associated with reduced occasional cocaine use but no such association was seen for regular cocaine use. This suggests that higher levels of cocaine use in methadone-maintained patients may not be influenced by medical follow-up for opioid dependence.

Some limitations of this study have to be acknowledged. First, we did not identify cocaine dependence in our sample. This data would have been interesting to analyze as cocaine-dependent patients may have been those who did not respond to MMT. The role of cocaine use in ADHD should be investigated more thoroughly, as the ASRS-v1.1 recently reported low specificity in the detection of ADHD among populations with substance use disorders [56]. The second limitation is that the validity of self-reported behaviors is often questioned due to the risk of underreporting linked to social desirability bias. However, this effect modifies OR estimates in a conservative manner. Furthermore, the reliability of self-reports based on using the Opiate Treatment Index (OTI) treatment questionnaire in drug-using populations has already been demonstrated [57].

## Conclusions

As suggested by previous findings, in our study, time on MMT had a positive impact on occasional cocaine users. However, regular cocaine use was not influenced by MMT duration but was associated with psychiatric comorbidities (ADHD and depression) and more severe addictive profiles (opioid injection). Early screening of these disorders and prompt tailored pharmacological and behavioral interventions have the potential to reduce cocaine use and to improve response to MMT.

## Abbreviations

ADHD: attention deficit/hyperactivity disorder
ASRS: Adult ADHD Self-Report Scale-Version
AUDIT: alcohol use disorders test
BHS: Beck Hopelessness Scale
CATI: computerized assisted phone interviews
CBT: cognitive behavioral therapy
CES-D: Center for Epidemiologic Studies-Depression
IQR: interquartile range
MMT: methadone maintenance treatment
OMT: opioid maintenance treatment
OOWS: Objective Opioid Withdrawal Scale
OTI: Opiate Treatment Index
